# Comparative Leaf and Root Transcriptomic Analysis of two Rice *Japonica* Cultivars Reveals Major Differences in the Root Early Response to Osmotic Stress

**DOI:** 10.1186/s12284-016-0098-1

**Published:** 2016-05-23

**Authors:** Elena Baldoni, Paolo Bagnaresi, Franca Locatelli, Monica Mattana, Annamaria Genga

**Affiliations:** Institute of Agricultural Biology and Biotechnology - National Research Council, via Bassini 15, 20133 Milan, Italy; Dipartimento di Scienze Agrarie e Ambientali - Produzione, Territorio, Agroenergia, Università degli Studi di Milano, Via Celoria 2, 20133 Milan, Italy; Consiglio per la ricerca in agricoltura e l’analisi dell’economia agraria, Genomics Research Centre, Fiorenzuola d’Arda, Piacenza Italy

**Keywords:** Hormones, Lignin, *Oryza sativa*, Osmotic stress, RNA-Seq analysis, Transcription factors

## Abstract

**Background:**

Rice (*Oryza sativa* L.) is one of the most important crops cultivated in both tropical and temperate regions and is characterized by a low water-use efficiency and a high sensitivity to a water deficit, with yield reductions occurring at lower stress levels compared to most other crops. To identify genes and pathways involved in the tolerant response to dehydration, a powerful approach consists in the genome-wide analysis of stress-induced expression changes by comparing drought-tolerant and drought-sensitive genotypes.

**Results:**

The physiological response to osmotic stress of 17 *japonica* rice genotypes was evaluated. A clear differentiation of the most tolerant and the most sensitive phenotypes was evident, especially after 24 and 48 h of treatment. Two genotypes, which were characterized by a contrasting response (tolerance/sensitivity) to the imposed stress, were selected. A parallel transcriptomic analysis was performed on roots and leaves of these two genotypes at 3 and 24 h of stress treatment. RNA-Sequencing data showed that the tolerant genotype Eurosis and the sensitive genotype Loto mainly differed in the early response to osmotic stress in roots. In particular, the tolerant genotype was characterized by a prompt regulation of genes related to chromatin, cytoskeleton and transmembrane transporters. Moreover, a differential expression of transcription factor-encoding genes, genes involved in hormone-mediate signalling and genes involved in the biosynthesis of lignin was observed between the two genotypes.

**Conclusions:**

Our results provide a transcriptomic characterization of the osmotic stress response in rice and identify several genes that may be important players in the tolerant response.

**Electronic supplementary material:**

The online version of this article (doi:10.1186/s12284-016-0098-1) contains supplementary material, which is available to authorized users.

## Background

Drought is one of the most important environmental constraints affecting plant growth and development and ultimately leads to yield loss. Water deficiency is a global concern because even the most productive agricultural regions can occasionally experience short periods or years of severe drought. Furthermore, irrigation might be restricted in the future because of the competition from other non-agricultural sectors, such as industry and urban areas (Bouman et al. 2007). Moreover, drought will continue to become worse in the next decades because of the potential impact of climate change on rainfall patterns and the need to extend the exploitation of marginal lands (Bates et al. 2008). Therefore, the implementation of water management practices and the development of both drought-tolerant varieties and water-use-efficient crops are key strategies to maintain yields under climate change conditions, extend cultivation to sub-optimal cropping areas and save water for sustainable agriculture. However, the development of drought-tolerant varieties still represents a challenging task, being hampered by the occurrence of genotype × environment interactions, the difficulty of effective drought tolerance screening and a still partial understanding of the molecular mechanisms of plant drought tolerance (Richards 1996; Kumar et al. 2008).

When plants perceive stress signals from their surroundings, a number of physiological, biochemical and molecular modifications occur (Krasensky and Jonak 2012). Some of these responses merely represent a consequence of cell damage, while others correspond to adaptive processes plants have evolved to cope with environmental cues. At the molecular level, the expression of a large number of genes is modulated under water stress conditions (Shinozaki and Yamaguchi-Shinozaki 2007; Qin et al. 2011; Yoshida et al. 2014). These genes encode either proteins with a direct role in protecting cell structures (e.g.*,* metabolic enzymes, late embryogenesis-abundant proteins, detoxification enzymes and chaperones), or proteins with a regulatory function (e.g.*,* transcription factors (TFs), protein kinases and other proteins involved in signal transduction) (Valliyodan and Nguyen 2006; Shinozaki and Yamaguchi-Shinozaki 2007; Hadiarto and Tran 2011). In particular, the identification of genes and pathways involved in the tolerant response to dehydration is clearly a crucial step in the development of drought-tolerant varieties. A powerful approach, which is increasingly being used to discriminate between drought tolerance-related genes and drought-responsive genes, is to perform genome-wide analyses of stress-induced expression changes by comparing drought-tolerant and drought-sensitive genotypes, rather than performing gene expression experiments on single genotypes (Moumeni et al. 2011; Utsumi et al. 2012; Guimaraes et al. 2012; Degenkolbe et al. 2013). This approach has allowed for the identification of genes with a positive function in enhancing drought tolerance and is potentially useful for the development of molecular markers to accelerate breeding programs.

Rice (*Oryza sativa* L.) is one of the most important crops cultivated in both tropical and temperate regions, representing the staple food for a large fraction of the world population. Rice is a high water demanding species, using approximately 40 % of the water diverted for irrigation (Lampayan et al. 2015), and rice cultivation is characterized by a low water-use efficiency and a high sensitivity to water deficit, with yield reductions occurring at lower stress levels compared to most other crops. Rice cultivation relies on cropping systems based on different water regimes, from irrigated systems to rainfed lowland and upland rice fields to deep water fields. The increasingly frequent occurrence of drought and the possible future restrictions of water availability for agricultural purposes are among the major challenges to be met to achieve sustainable rice production. Actually, it is estimated that by 2025, 15 to 20 million hectares of irrigated rice fields will suffer from some degree of water scarcity (Lampayan et al. 2015). For these reasons, the development of new rice cultivars with a better water-use efficiency or an enhanced drought tolerance is a primary goal in rice breeding programs. Currently, an increasing number of studies focuses on the identification of drought responsive genes that are differentially regulated in rice genotypes characterized by a contrasting phenotype in response to stress (Degenkolbe et al. 2009; Lenka et al. 2011; Cal et al. 2013; Degenkolbe et al. 2013; Moumeni et al. 2015).

In the present work, a parallel transcriptomic analysis was conducted on two Italian rice genotypes characterized by a contrasting phenotype in response to osmotic stress. RNA-Sequencing was performed separately on leaves and roots to characterize the specific response of these organs in the considered genotypes. The results of this study may contribute to elucidating the mechanisms involved in the rice response to osmotic stress and to identify genes that are putatively responsible for the stress-tolerant phenotype.

## Results and Discussion

### Physiological Response to Osmotic Stress

To evaluate the physiological response to osmotic stress of 17 *japonica* rice cultivars, which are currently listed in the Italian National Register, the leaf relative water content (RWC; Table 1) and the leaf electrolyte leakage (EL; Table 2) of plants subjected to 0, 3, 24 and 48 h of 20 % polyethylene glycol (PEG) treatment were measured. The rice cultivars showed different responses to the imposed stress. After 24 and 48 h of treatment, a clear differentiation of the most tolerant and the most sensitive phenotypes was evident. In particular, after 48 h of treatment, Carnaroli, Gigante Vercelli, Loto, Maratelli and Vialone Nano resulted to be the most sensitive cultivars, showing both the lowest RWC (<15 %) and the highest EL (>94 %) values, whereas Augusto and Eurosis resulted to be the most tolerant genotypes, showing both the highest RWC values (>80 %) and the lowest EL values (<40 %). The other cultivars (Thaibonnet, Baldo, Gladio, Koral, Salvo, SISR215, Volano, Arborio, Venere, Asia) exhibited an intermediate phenotype. In a previous study, we investigated the response to osmotic stress of 8 Italian rice cultivars; among them, 6 cultivars analyzed in the present work (Arborio, Augusto, Baldo, Eurosis, Loto and Vialone Nano) were included (Baldoni et al. 2013). In that analysis, Augusto and Eurosis resulted among the most tolerant cultivars, with RWC values > 60 % after 48 h of treatment, whereas Loto and Vialone Nano resulted the most sensitive cultivars, with RWC values < 15 % after 48 h of treatment. The present work, in which a higher number of cultivars was analyzed, confirmed the highly tolerant phenotype for Augusto and Eurosis, and the highly sensitive phenotype for Loto and Vialone Nano in response to osmotic stress.

**Table 1:** Relative water content (RWC) measurements of rice cultivars

	start		3 h		24 h		48 h	
CV	mean	*p*	mean	*p*	mean	*p*	mean	*p*
Carnaroli	95.3	bcd	68.3	abcd	16.8	abc	14.3	a
Gigante Vercelli	94.4	abcd	66.9	abc	17.2	abc	12.0	a
Loto	91.8	a	64.6	ab	33.9	abcde	11.7	a
Maratelli	92.9	ab	63.4	a	13.7	ab	13.8	a
Vialone Nano	93.3	abc	62.8	a	11.9	a	9.2	a
Thaibonnet	96.7	d	84.0	efg	39.7	cdef	18.5	ab
Baldo	94.6	abcd	78.1	defg	35.7	bcdef	23.6	abc
Gladio	97.0	d	85.6	fg	28.1	abcd	31.5	abc
Koral	92.9	ab	77.9	defg	50.6	defg	27.0	abc
Salvo	96.2	cd	76.8	cdef	30.3	abcde	28.1	abc
SISR215	95.3	bcd	78.4	defg	37.1	cdef	30.4	abc
Volano	93.9	abcd	74.7	bcde	34.4	abcdef	26.7	abc
Arborio	95.7	bcd	79.8	efg	52.8	efg	37.6	bc
Venere	96.4	cd	87.9	g	62.9	gh	44.6	cd
Asia	96.1	cd	85.3	efg	57.0	fgh	63.5	de
Augusto	94.3	abcd	76.9	cdef	76.6	hi	82.4	ef
Eurosis	94.2	abcd	87.0	fg	89.7	i	86.4	f

Data were taken following 0, 3, 24 and 48 h of PEG treatment. Each percentage value is the mean of 5 plants. For each sampling time, data were subjected to a one-way analysis of variance to compare the different varieties. Different letters in the same column show significant differences based on a Tukey’s test (*p* ≤ 0.001)

**Table 2:** Electrolyte leakage (EL) measurements of rice cultivars

	Start		3 h		24 h		48 h	
CV	Mean	*p*	Mean	*p*	Mean	*p*	Mean	*p*
Gigante vercelli	18.6	cd	41.8	h	94.8	lm	97.2	h
Carnaroli	16.4	abc	23.8	abc	88.0	hi	95.6	gh
Loto	16.7	bc	49.5	i	89.7	il	94.4	gh
Maratelli	13.6	a	42.0	h	95.8	m	95.8	gh
Vialone Nano	24.4	fg	40.2	gh	84.8	hi	96.2	gh
Volano	25.0	g	36.2	g	83.4	h	94.0	gh
SISR215	21.6	ef	31.2	f	56.2	c	91.6	fgh
Thaibonnet	22.0	ef	30.0	ef	67.4	f	89.8	fg
Baldo	14.6	ab	21.2	a	65.0	ef	85.8	ef
Koral	20.0	de	28.5	def	66.7	ef	80.3	de
Salvo	15.8	abc	22.6	ab	61.6	de	81.2	de
Gladio	14.2	ab	22.2	ab	76.0	g	78.0	d
Venere	26.0	g	32.0	f	52.8	c	69.8	c
Arborio	21.8	ef	30.6	ef	56.6	cd	58.4	b
Asia	16.8	bc	23.6	abc	43.8	b	55.0	b
Augusto	18.2	cd	25.4	bcd	27.8	a	30.6	a
Eurosis	21.6	ef	27.0	cde	29.0	a	35.8	a

Data were taken following 0, 3, 24 and 48 h of PEG treatment. Each percentage value is the mean of 3 plants. For each sampling time, data were subjected to a one-way analysis of variance to compare the different varieties. Different letters in the same column show significant differences based on a Tukey’s test (*p* ≤ 0.001)

Based on the obtained physiological results and on the rice grain type classification, Eurosis and Loto (both Long A grain rice cultivars) were selected as tolerant and sensitive genotypes, respectively, for transcriptome analysis, to identify differentially expressed genes (DEGs) and differentially regulated pathways under osmotic stress.

### RNA-Seq Data Analysis and Evaluation of DEGs

**Fig. 1:**
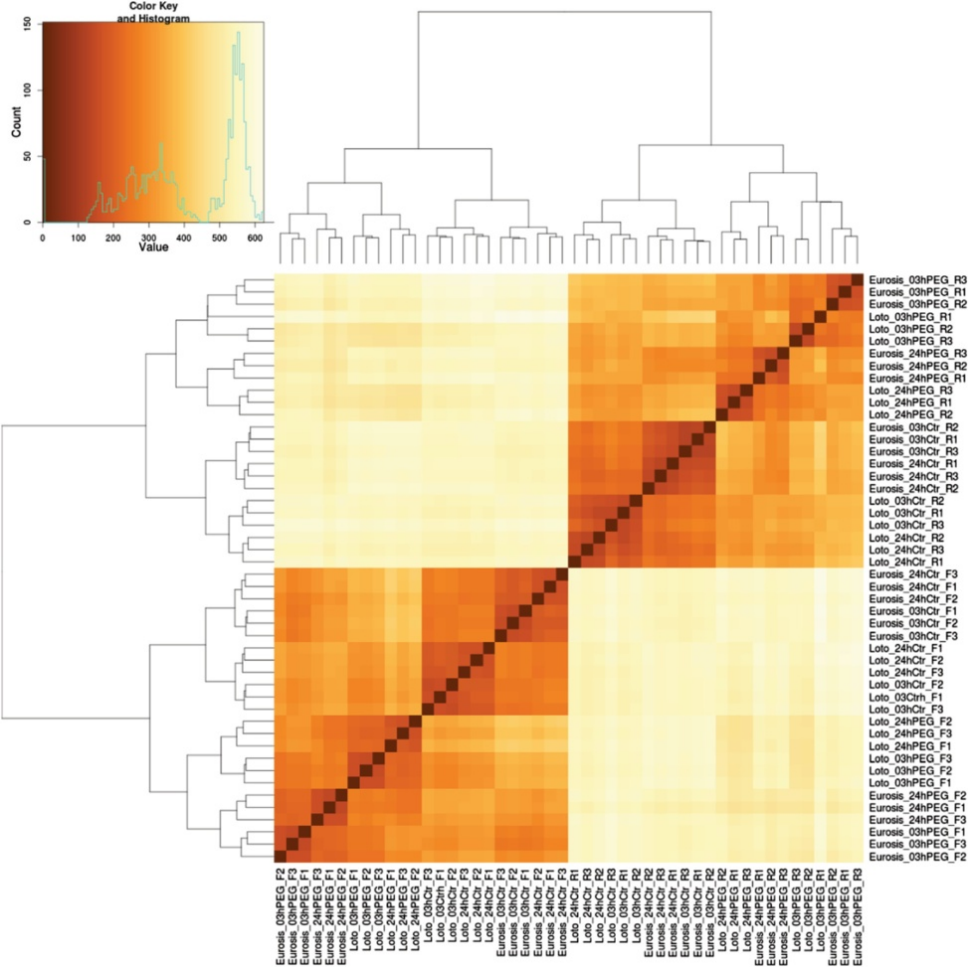
Clustering and heatmaps of samples. DESeq-normalized count data were transformed with the VST function (DESeq package). Eurosis and Loto (control/Ctr or osmotic/PEG treatment) samples and biological replicates (F1, F2 and F3 for leaves, and R1, R2 and R3 for roots) are shown. The colour scale indicates the degree of correlation (*white-yellow, low correlation; orange-red, strong correlation*)

RNA samples that were isolated from leaves and roots of Eurosis and Loto genotypes at 3 and 24 h of osmotic stress treatment were subjected to whole transcriptome sequencing, to analyze the short-term response to osmotic stress. The corresponding control samples were also analysed. Three biological replicates were performed for each rice genotype and condition (48 samples in total). Raw reads (50 bases, single-end) obtained from Illumina HiSeq sequencing were filtered (Illumina passed-filter call), and the fastQC application was employed to detect sequence contaminants. Contaminant-free, filtered reads (trimmed to 43 bases to discard low-quality 3′ terminal regions) ranging from 17 to 36 million per sample (Additional file 1: Table S1) were mapped with Bowtie 0.12.7 and TopHat 1.4.1 to the rice Nipponbare genome (Ensembl plants release MSU 6.16). Raw read counts were obtained from BAM alignment files by counting with HTSeq software. An RPKM (Reads per Kilobase per Million) cut-off value of 0.1 was set to declare a locus expressed resulting in 32,862 and 32,778 *loci* above the expression cut-off for Eurosis and Loto varieties, respectively. All of the biological replicates exhibited Pearson correlation coefficients above 0.9, indicating a good level of reproducibility among biological replicates (Fig. 1 and Additional file 2: Table S2).

To identify DEGs, the R package DESeq was employed. Two countset instances were created for Eurosis and Loto. The false discovery rate (FDR) threshold was set to 0.001, and gene dispersion values were calculated using the “maximum” mode. Given the large number of DEGs detected in all of the contrasts, a threshold of 3-fold expression change was further established to focus on a subset of DEGs showing highest modulation.

**Fig. 2:**
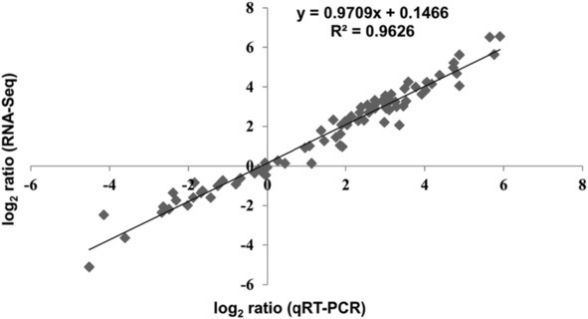
Validation of the expression of selected genes from RNA-Seq using qRT-PCR. Fold changes in gene expression were transformed to a log_2_ scale. The qRT-PCR data log_2_-values *(X-axis*) were plotted against the RNA-Seq log_2_ values (*Y-axis*). The function of the regression line and the R^2^ value are shown

Gene expression changes for 10 selected genes, that were identified as DEGs during the RNA-Seq experiments and were reported to be involved in the osmotic stress response, were validated using qRT-PCR. The comparison between RNA-Seq and qRT-PCR fold change data, which was obtained by a regression analysis, revealed a substantial agreement in the extent of the osmotic stress-induced variations in transcript accumulation for the 10 tested genes (Fig. 2 and Additional file 3: Table S3). The transcript levels of 6 of these genes (LOC_Os01g07120, LOC_Os01g66120, LOC_Os02g44870, LOC_Os04g43680, LOC_Os04g45810 and LOC_Os10g33810) have been previously analysed using qRT-PCR in rice samples of Eurosis and Loto plants under the same stress conditions (Baldoni et al. 2013). The comparison between qRT-PCR data presented here and in the previous work revealed comparable stress-induced fold changes, indicating a good reproducibility of the osmotic stress experiments (data not shown).

**Fig. 3:**
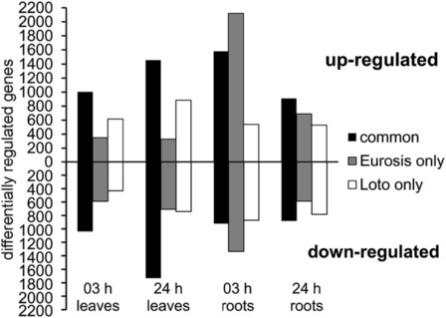
Up- and down-regulated genes in leaf or root samples at 3 and 24 h of PEG treatment. *Black bars*: common regulated genes in the two cultivars. *Grey bars*: Eurosis specific up- or down-regulated genes. *White bars*: Loto specific up- or down-regulated genes

**Fig. 4:**
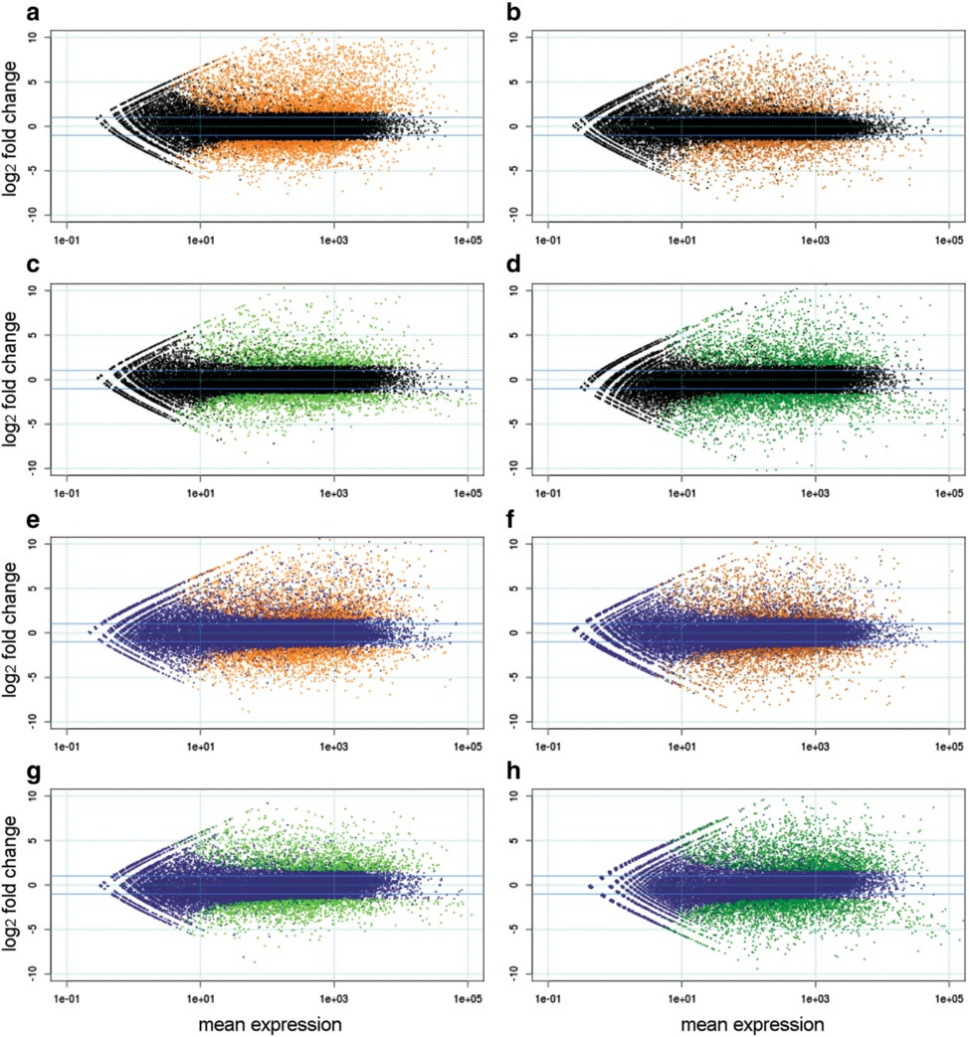
Mean expression vs. log fold change plots (MA-plots) of Eurosis and Loto samples. Mean expression vs. log fold change plots (MA-plots) were computed for Eurosis (*plotted in black*; **a**-**d**) and Loto (*plotted in blue*; **e**-**h**) contrasts. Normalised expression mean values are plotted vs. log_2_fold changes. Called DEGs (FDR < 0.001 and fold change > 3) are plotted in orange for root samples (**a**, **b**, **e** and **f**) and in green for leaf samples (**c**, **d**, **g** and **h**)

The RNA-Seq analysis showed that osmotic stress caused significant changes in gene expression in the roots and leaves of both genotypes (Fig. 3, Table 3 and Additional file 4: Table S4). In particular, 3 h of osmotic stress led to a substantial modulation of gene expression in the roots of the tolerant genotype Eurosis (6007 genes), whereas fewer DEGs were called in the roots of the susceptible genotype Loto (3962 genes). At 24 h of treatment, a similar number of DEGs was found in Eurosis and Loto roots (3065 and 3102 genes, respectively). In the leaves, a similar number of DEGs was found in the two genotypes, both at 3 and 24 h of osmotic stress treatment (2977 and 4223 in Eurosis, and 3088 and 4813 in Loto, respectively) (Table 3). The stress response of the two cultivars consisted of a common and a specific component (Fig. 3 and Table 3). In particular, the specific response was prominent in the roots. Indeed, in the roots at 3 h of stress treatment, 2499 DEGs (representing only 34 % of the total DEGs in this tissues/time of treatment) were common to Eurosis and Loto, whereas 66 % were specific to one or the other genotype (47 and 19 % to Eurosis and Loto, respectively). Only 53 DEGs (0.7 %) were oppositely modulated in the two genotypes in this tissue/time of treatment. It is noteworthy that in all of the other contrasts, no oppositely modulated DEGs were observed. In 24 h-treated roots, a proportion of 41 % of the DEGs was common to Eurosis and Loto, whereas 59 % were specific to one or the other genotype. In the leaves, the proportion of common DEGs was higher than in the roots, with 51 and 54 % at 3 and 24 h of stress treatment, respectively (Fig. 3 and Table 3). Regarding up-and down-regulated genes, the mean expression vs. logfold change plots (MA-plots, Fig. 4) illustrated that, among the DEGs, the proportion of up- and down-regulated genes was substantially similar in all the control vs. treated comparisons, with the exception of the 3 h root samples of Eurosis, in which the number of up-regulated genes (61.6 % of all DEGs) was higher than the number of down-regulated genes (38.4 %). In summary, major differences between the responses of the two cultivars were observed at 3 h of osmotic treatment in roots, where a higher number of genes were significantly regulated and where the common response was less represented than in the other tested conditions.

**Table 3:** Summary of the total number of DEGs, DEGs common to both cultivars and cultivar-specific DEGs

Tissue	Contrasts	Total number of DEGs		Common DEGs	Specific DEGs	
		Eurosis	Loto		Eurosis	Loto
Roots	3 h control vs. 3 h stress	6007	3962	2552^a^	3455	1410
Roots	24 h control vs. 24 h stress	3065	3102	1789	1276	1313
Leaves	3 h control vs. 3 h stress	2977	3088	2040	937	1048
Leaves	24 h control vs. 24 h stress	4223	4813	3187	1036	1626

^a^Among the 2552 common DEGs, 53 genes were oppositely regulated between the 2 genotypes, whereas 2499 were modulated in the same manner in the 2 genotypes

As mentioned above, DEGs showing an opposite modulation between the two genotypes were found only in 3 h-treated roots; that is, 4 DEGs were up-regulated in Eurosis and down-regulated in Loto, and 49 DEGs were up-regulated in Loto and down-regulated in Eurosis (Table 3 and Additional file 5: Table S5). Thus far, no literature data are available on the function of the 4 genes that were up-regulated in Eurosis and down-regulated in Loto; only little information is present in the literature about some of the 49 genes that were up-regulated in Loto and down-regulated in Eurosis. In particular, LOC_Os01g06500 codes for a lectin-like protein, and its expression correlated with the isoprenoid biosynthetic pathway (Suzuki et al. 2007). In rice, the salt-inducible gene *SalT* encodes a cytoplasmic mannose-binding lectin, whose expression increases after salt or drought stress (de Souza Filho et al. 2003). In general, the expression of lectin-encoding genes may be regulated under abiotic or biotic stress conditions, and a putative role for these genes in plant stress response has been proposed (Rüdiger and Gabius 2001; Van Damme et al. 2004). Moreover, among these 49 genes, some putative TF-encoding genes were present, namely, 1 MYB (LOC_Os02g17190), three putative PHD finger family proteins (LOC_Os03g53630, LOC_Os11g29240 and LOC_Os12g24540) and one putative bZIP (LOC_Os03g20530). These genes may be involved in the upstream regulation of the osmotic stress response and may have a crucial role in establishing a different response to the imposed stress in the two genotypes.

### Gene Ontology Enrichment Analysis of RNA-Seq Data

Gene ontology (GO) enrichment analysis of DEGs in the various contrasts was conducted to reveal biological trends differentiating tolerant and susceptible genotypes. The goseq bioconductor package (Robinson and Oshlack 2010) was used to account for the RNA length bias that is typical of RNA-Seq approaches (Oshlack and Wakefield 2009). Both common and genotype-specific enriched terms were identified, confirming the presence of a consistent common response to osmotic stress and genotype-specific responses, as observed in the analysis of DEGs (Fig. 3).

#### Common Enriched GO Terms

Sixty-one enriched GO terms were shared by the 2 rice genotypes in all of the analysed tissues and treatments (Additional file 6: Table S6). Among the 21 GO terms related to Biological Processes (BP), some were consistent with responses to water/osmotic stress; these included “response to water deprivation” (GO:0009414), “response to water stimulus” (GO:0009415) and “response to stress” (GO:0006950). Moreover, several common GO terms were related to the response to abiotic stresses, such as “response to oxidative stress” (GO:0006979), “response to cold” (GO:0009409), “response to wounding” (GO:0009611), “response to salt stress” (GO:0009651) and “response to freezing” (GO:0050826). This result was not unexpected because the existence of cross-talk among pathways involved in the response to different abiotic stresses is well known (Qin et al. 2011; Nakashima et al. 2014).

Moreover, the GO terms “response to abscisic acid stimulus” (GO:0009737) and “cellular response to abscisic acid stimulus” (GO:0071215) were shared by all of the samples, and this is consistent with the well known pivotal role of abscisic acid (ABA) signalling in the response to osmotic stress (Fujita et al. 2011). Additional GO terms related to hormone signalling were shared by all of the root samples but not by leaf samples, or vice-versa; in the roots, two GO terms were related to salicylic acid and jasmonic acid-mediated responses (GO:0009751 and GO:0009753, respectively), whereas the GO terms “response to ethylene stimulus” (GO:0009723) and “indolebutyric acid metabolic process” (GO:0080024) were shared only by the leaf samples.

The enriched GO terms shared by all of the root samples or by all of the leaf samples were 20 and 70, respectively, confirming a prominent common response in leaves. Water stress highly affects the photosynthetic processes (Chaves et al. 2009). As expected, many enriched GO terms common to leaf samples were related to photosynthesis (Additional file 6: Table S6). Because of the inhibition of photosynthesis during stress, plants must mobilize energy from storage resources, such as carbohydrates, fatty acids and proteins (Shu et al. 2011). Actually, some GO terms related to fatty acid metabolism were shared by leaf samples of the 2 genotypes, namely, “lipid metabolic process” (GO:0006629), “fatty acid biosynthetic process” (GO:0006633) and “lipid biosynthetic process” (GO:0008610) (Additional file 6: Table S6).

#### Genotype-Specific Enriched GO Terms

**Fig. 5:**
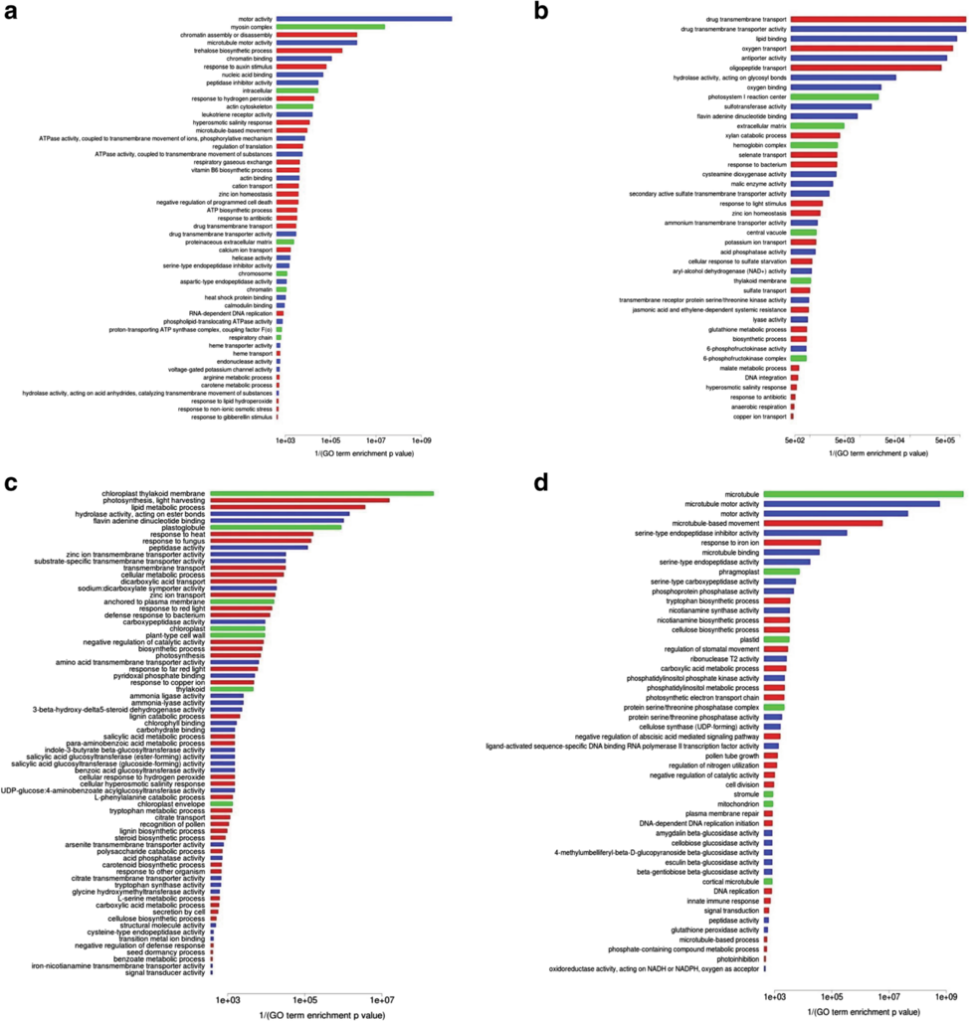
Barplots of differentially enriched GO terms in the roots. Barplots of differentially enriched GO terms (from one genotype/organ/treatment missing in the alternative genotype in the same organ/treatment) in the roots, as estimated by goseq at an FDR cut-off of 0.05. Reciprocal of enrichment *p*-values are shown on the X axis. Red boxes indicate Biological Process GO terms; blue boxes indicate Molecular Function GO terms; green boxes indicate Cell Compartment GO terms. **a** Eurosis 3 h; **b** Eurosis 24 h; **c** Loto 3 h; **d** Loto 24 h

**Fig. 6:**
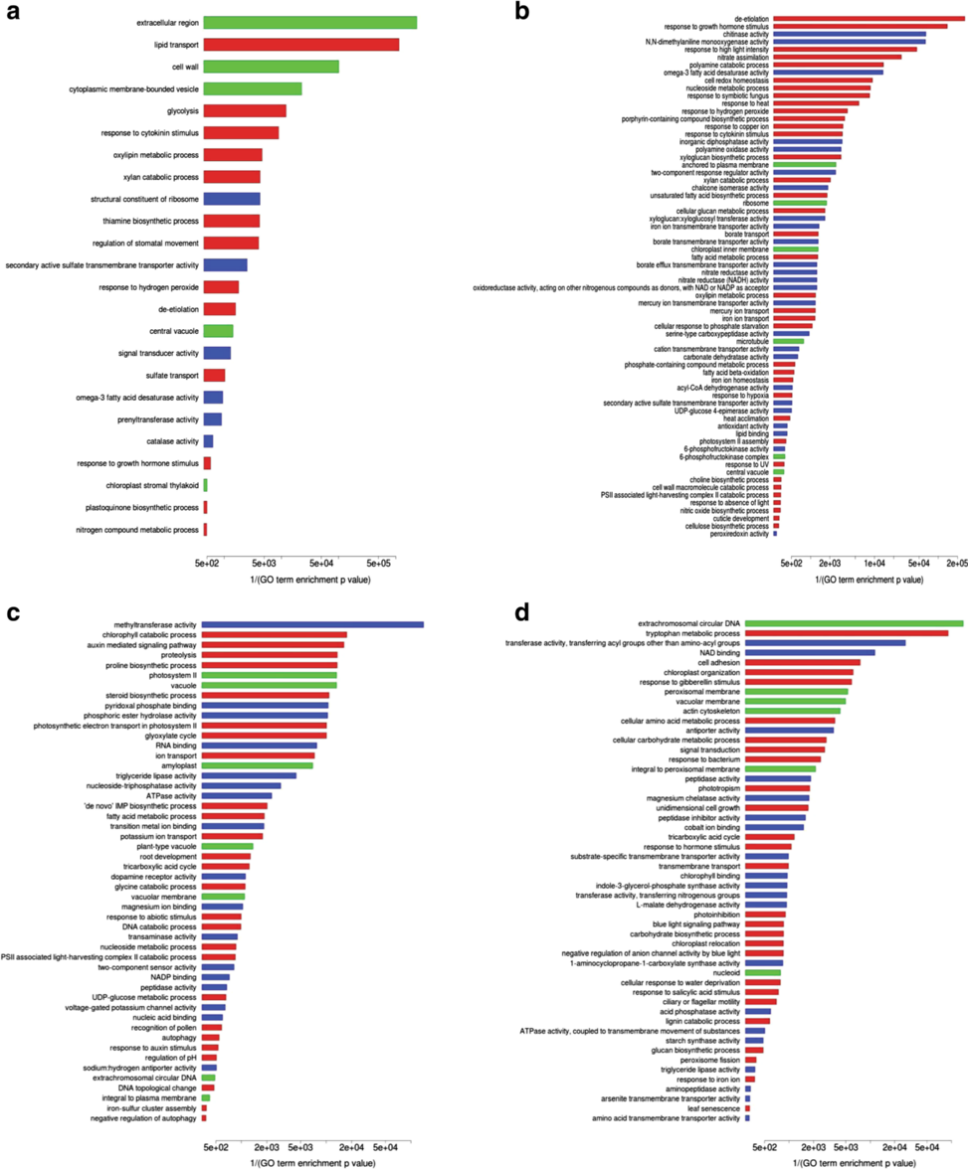
Barplots of differentially enriched GO terms in the leaves. Barplots of differentially enriched GO terms (from one genotype/organ/treatment missing in the alternative genotype in the same organ/treatment) in the leaves, as estimated by goseq at an FDR cut-off of 0.05. Reciprocal of enrichment *p*-values are shown on the X axis. Red boxes indicate Biological Process GO terms; blue boxes indicate Molecular Function GO terms; green boxes indicate Cell Compartment GO terms. **a** Eurosis 3 h; **b** Eurosis 24 h; **c** Loto 3 h; **d** Loto 24 h

Several GO terms were genotype-specific (i.e.*,* enriched GO terms from one genotype/organ/treatment missing in the alternative genotype in the same organ/treatment). Additional file 7: Table S7 reports all of the enriched GO terms for the contrasts with accompanying enrichment *p*-values resulting from goseq analysis. Figures 5 and 6 depict genotype-specific barplots. Because DEG analysis found that the genotype-specific response was prominent in roots, our analysis mainly focused on root samples.

#### Stress and Hormone-Related GO Terms

Some enriched GO terms specific to either the Eurosis or the Loto genotype were related to osmotic stress response, i.e.*,* “trehalose biosynthetic process” (GO:0005992), “response to non-ionic osmotic stress” (GO:0010335) and “hyperosmotic salinity response” (GO:0042538) in Eurosis roots (Fig. 5a and b); “cellular hyperosmotic salinity response” (GO:0071475) in Loto roots (Fig. 5c); “choline biosynthetic process” (GO:0042425) in Eurosis leaves (Fig. 6b); “proline biosynthetic process” (GO:0006561), “response to abiotic stimulus” (GO:0009628) and “cellular response to water deprivation” (GO:0042631) in Loto leaves (Fig. 6c and d). Some genes belonging to these GO terms may play a role in the different response of the two genotypes. Among them, LOC_Os03g04570, which was up-regulated only in Eurosis 3 h-treated roots and belonged to the GO term GO:0042538 (Additional file 7: Table S7), codes for a putative peptide transporter (PTR). The PTR family consists of di/tripeptides transporters, and their role in plants is not yet well defined (Saier et al. 2006). This gene is similar to *AtPTR3* (AT5G46050), whose expression is induced by salt stress and mechanical wounding and is regulated by jasmonic acid and salicylic acid (Karim et al. 2005; Karim et al. 2007). Another interesting gene is LOC_Os07g01020, which was up-regulated in Eurosis 3 h- and 24 h-treated roots and in Loto 24 h-treated roots and belonged to the GO terms GO:0010335 and GO:0042538 (Additional file 7: Table S7). This gene codes for a putative SOR/SNZ family protein that has high homology with the pyridoxine synthase gene AT5G01410 (Chen et al. 2014). Interestingly, Jeong et al. (2013) observed that in transgenic rice plants over-expressing *OsNAC5* under the control of a root-specific promoter, LOC_Os07g01020 was up-regulated together with other genes implicated in root growth and development. These plants showed a higher grain yield under drought conditions compared to wild-type plants and an increased root diameter, which could contribute to the enhanced drought tolerance. In our work, the specific induction of LOC_Os07g01020 in Eurosis 3 h-treated roots suggests a possible role for this gene in the early response to osmotic stress of this genotype.

Moreover, several enriched GO terms specific to Eurosis or Loto were related to hormone metabolism (Figs. 5 and 6, Additional file 7: Table S7). Some genes belonging to these GO terms may have a role in the different responses of the two genotypes. For instance, a *MYB* gene (LOC_Os01g34060) belonging to GO terms related to the response to auxin and gibberellin stimulus (GO:0009733 and GO:0009739, respectively) was up-regulated in Eurosis roots after 3 h of treatment and in Loto roots after 24 h, suggesting a delay in the Loto response (Additional file 7: Table S7).

#### Chromatin-Related GO Terms

Among GO terms specifically enriched in 3 h-treated roots of the tolerant genotype Eurosis, 5 were associated with chromatin (Fig. 5a and Additional file 7: Table S7), namely, “chromatin” (GO:0000785), “chromatin binding” (GO:0003682), “helicase activity” (GO:0004386), “chromosome” (GO:0005694) and “chromatin assembly or disassembly” (GO:0006333). The enrichment of these GO terms was due to the statistically significant modulation of 71 genes (12 up- and 59 down-regulated), which occurred exclusively in Eurosis. Notably, only 1 out of the up-regulated genes and 3 out of the down-regulated genes were listed among the DEGs in the 24 h-treated root samples, suggesting that the transcriptomic response associated with chromatin-related processes is an early and transient response in the tolerant cultivar Eurosis.

Globally, the most represented protein family encoded by DEGs annotated within chromatin-related GO categories was the helicase protein superfamily. Helicases are molecular motors that mainly use the energy derived from ATP hydrolysis to bind, unwind or remodel energetically stable double-stranded DNA (DNA helicases) or local RNA secondary structures (RNA helicases). They are central players in virtually all facets of nucleic acid metabolism, and may play essential roles in the response to stress conditions (Vashisht and Tuteja 2006; Tuteja et al. 2013; Zhu et al. 2015).

In rice, 40 Snf2 proteins, which are ATP-dependent chromatin remodelling factors, have been identified, and some of these proteins were affected by stress treatments (Hu et al. 2013). In our 3 h-treated root dataset, 19 out of the 40 *Snf2* genes were listed among the DEGs. Among them, 3 genes were up-regulated exclusively in Loto, whereas the other 16 were down-regulated (14 only in Eurosis, 1 only in Loto and 1 in both genotypes). One of the genes specifically down-regulated in Eurosis 3 h-treated roots was LOC_Os06g08480, which is annotated as the CHD3-type chromatin-remodelling factor PICKLE. Interestingly, a role of the PICKLE factor in balancing osmotic stress responses during seed germination has been well demonstrated in Arabidopsis (Perruc et al. 2007). Li et al. (2011) found that LOC_Os06g08480 and another down-regulated *Snf2* gene in Eurosis 3 h-treated roots, namely, LOC_Os01g44990, were induced by several abiotic stresses, including PEG-mediated osmotic treatment, in the sensitive cultivar Zhonghua11.

Another represented protein family encoded by DEGs that are annotated within chromatin-related GO categories was the histone protein family. Notably, in our dataset, 7 DEGs annotated as histone proteins were exclusively up-regulated in Eurosis, and 1 was down-regulated in Loto. Recent studies have reported that chromatin regulation, and, in particular, histone modification and DNA methylation, are key elements of the transcriptional response to abiotic stresses, such as water deficit, high-salinity, and temperature shifts. However, it still remains unclear how transcriptional changes and chromatin changes are linked (Kim et al. 2015).

Among the DEGs, which are annotated with chromatin-related GO terms, 2 showed an opposite regulation in the two cultivars, LOC_Os02g04050 and LOC_Os02g50370, both being down-regulated in Eurosis and up-regulated in Loto (Additional file 4: Table S4). The former is annotated in MSU 6.16 as a putative chromosome segregation protein and the latter as a putative helicase domain-containing protein (Additional file 4: Table S4). To our knowledge, no function in response to any abiotic stress has been reported for these two genes.

It is currently understood that the regulation of abiotic stress responsive genes is related to chromatin alterations. Consistently, our results suggest that chromatin remodelling processes occur in response to osmotic stress and may contribute to differentiating the response between the tolerant and the sensitive cultivar.

#### Cytoskeleton-Related GO Terms

In 3 h-treated root samples, 6 GO categories specifically enriched in the Eurosis genotype were related to the cytoskeleton (Fig. 5a and Additional file 7: Table S7), namely, “motor activity” (GO:0003774), “microtubule motor activity” (GO:0003777), “actin binding” (GO:0003779), “microtubule-based movement” (GO:0007018), “actin cytoskeleton” (GO:0015629) and “myosin complex” (GO:0016459). A total of 57 DEGs (21 up-regulated and 36 down-regulated) were responsible for the enrichment of these GO terms in Eurosis 3 h-treated roots. Their expression did not significantly change in Loto 3 h-treated roots. Among the 36 down-regulated DEGs, several genes are annotated either as kinesins (16 genes) or myosins (9 genes), and few additional genes encode actin regulatory proteins, such as formin, villin, and SCAR-like proteins. Notably, 11 out of these 36 genes were significantly down-regulated in Loto roots after 24 h of treatment, thus suggesting a delay in the response of the sensitive cultivar in comparison to the tolerant one.

Interestingly, some genes associated with these GO terms showed an opposite regulation in the 3 h-treated roots of the two genotypes, all of them exhibiting a down-regulation in Eurosis and an up-regulation in Loto. Among them, two genes belong to the myosin gene family, namely, LOC_Os01g51632 and LOC_Os01g51634. One additional gene, LOC_Os03g06510, was annotated as KIP1. Kip-related proteins (KRPs) play a central role in the regulation of the cell cycle and differentiation through the modulation of cyclin-dependent kinases. In Arabidopsis, the overexpression of a member of the KRP family, KRP2, prevents pericycle activation and reduces the number of lateral roots, suggesting a significant role of KRP2 in the regulation of early lateral root initiation (Himanen et al. 2002). Another KRP protein, ICK3/KRP5, is a positive regulator of both cell growth and endoreduplication in roots (Wen et al. 2013). Some studies analysed the relationship between drought stress and the expression of the *KRP* genes involved in the reprogramming of cell proliferation and cell expansion in leaves (Claeys and Inze 2013; Guan et al. 2014). To our knowledge, no data are available in the literature on a correlation between the expression of *KRP* genes in roots and the tolerance to water stress.

Three additional genes, which showed an opposite regulation in the 3 h-treated roots of the two genotypes, belong to the kinesin gene family, namely, LOC_Os06g36080, LOC_Os07g44400 and LOC_Os09g25380. Kinesins are microtubule-based motor proteins that are ubiquitous in all eukaryotic organisms and use the energy derived from ATP hydrolysis to move along the cytoskeletal elements of microtubules. Kinesins are involved in microtubule organization, organelle and vesicle transport, cellulose microfibril order, and ultimately contribute to cell division, cell growth and the cross-talk of microtubules and actin microfilaments (Li et al. 2012). Recently, a kinesin-like calmodulin-binding protein has been reported to be involved in the signalling network that negatively regulates root growth in Arabidopsis (Humphrey et al. 2015). Considering the kinesin encoding DEGs that were genotype-specific in 3 h-treated roots, 16 were down-regulated in Eurosis and 6 were up-regulated in Loto. Notably, all of the kinesin encoding DEGs showed either a down-regulation in Eurosis or an up-regulation in Loto. This differential regulation of kinesin-encoding genes observed in 3 h-treated roots of Eurosis and Loto suggested that this protein family affects the root growth in a genotype-specific manner, thus contributing to the different osmotic stress phenotypes (tolerance/sensitivity) of the 2 cultivars.

#### Transmembrane Transport-Related GO Terms

Six enriched GO terms specific to the tolerant genotype Eurosis in the roots at 3 h of stress treatment were related to transmembrane transport (Fig. 5a and Additional file 7: Table S7), namely, “phospholipid-translocating ATPase activity” (GO:0004012), “drug transmembrane transport” (GO:0006855), “drug transmembrane transporter activity” (GO:0015238), “ATPase activity, coupled to transmembrane movement of ions, phosphorylative mechanism” (GO:0015662), “hydrolase activity, acting on acid anhydrides, catalyzing transmembrane movement of substances” (GO:0016820) and “ATPase activity, coupled to transmembrane movement of substances” (GO:0042626). Thirty-three genes were responsible for the enrichment of these GO terms in the tolerant cultivar. Among them, 10 genes were significantly up-regulated only in Eurosis 3 h-treated roots, including 7 genes encoding multidrug and toxic compound extrusion (MATE) family proteins and 2 genes encoding ATP-binding cassette (ABC) transporter family proteins, whereas 23 genes were specifically down-regulated in Eurosis 3 h-treated roots, including 12 genes encoding ABC transporter family proteins. ABC and MATE proteins represent the two largest families of active transporters that have been characterized in plants thus far. ABC proteins are primary transporters that mediate the energy-driven transport of a large and diverse multitude of substrates across biological membranes. ABC transporters play key roles in many physiological processes, such as plant development and plant nutrition, and in stress response (Moons 2008; Matsuda et al. 2012; Jarzyniak and Jasinski 2014; Nguyen et al. 2014; Saha et al. 2015). In the rice genome, 133 genes coding for ABC transporters have been identified (Saha et al. 2015). In our study, in 3 h-treated roots, 15 out of the 133 ABC transporter genes were modulated in the same way in the two cultivars, 4 and 7 ABC transporter genes were specifically up-regulated in Eurosis and Loto, respectively, and 14 and 3 ABC transporter genes were specifically down-regulated in Eurosis and Loto, respectively. Among the 4 Eurosis-specific up-regulated genes, the *OsABCG22* gene (LOC_Os09g29660) is induced by mannitol and by ABA treatments in rice roots (Matsuda et al. 2012). Moreover, Nguyen et al. (2014) found that drought stress induced this gene in the leaves. Our results are consistent with these data, as this gene was also up-regulated in our leaf samples, both at 3 and 24 h of stress treatment.

In addition, the superfamily of MATE proteins represents one of the largest transporter families in plants, with more than 50 MATE genes in the rice genome. MATEs are secondary active transporters; in plants they have many physiological functions, such as the accumulation of secondary metabolites, aluminium detoxification and iron translocation (Takanashi et al. 2014; Jarzyniak and Jasinski 2014). Some MATEs are involved in hormone signalling in Arabidopsis, such as AtEDS5, which is responsible for intracellular salycilic acid transport, and AtDTX50, which functions as an ABA efflux transporter and plays a role in ABA-mediated growth inhibition and responses to drought conditions (Takanashi et al. 2014). In our study, among the 50 genes annotated as MATE efflux family proteins or MATE domain containing proteins, 8 DEGs (7 up- and 1 down-regulated) were specific to Eurosis 3 h-treated roots, and 2 DEGs (both down-regulated) were specific to Loto 3 h-treated roots. To our knowledge, no data are available in the literature on a putative role for any of these MATE efflux genes in water stress response. However, the observed stress-mediated regulation of several MATE genes suggests a possible role for some of them in the response to osmotic stress.

Finally, LOC_Os06g12876, which belongs to the GO terms GO:0016820, was 1 of the 49 genes oppositely modulated (down- and up-regulated in Eurosis and Loto, respectively) in 3 h-treated roots (Table 3). This gene is annotated in RAP-DB as an “ATPase, F1/V1/A1 complex, alpha/beta subunit”. Thus far, no data are available on the putative function of this gene.

#### Oxygen-Related GO Terms

In Eurosis 24 h-treated roots, 5 enriched GO terms were related to oxygen (Fig. 5b), namely, “response to hypoxia” (GO:0001666), “anaerobic respiration” (GO:0009061), “oxygen transport” (GO:0015671), “oxygen binding” (GO:0019825) and “haemoglobin complex” (GO:0005833). The rice genome contains five genes encoding non-symbiotic haemoglobins (ns-Hbs), namely *OsNSHB1* to *OsNSHB5* (LOC_Os03g13140, LOC_Os03g12510, LOC_Os03g13150, LOC_Os03g13160 and LOC_Os05g44140, respectively) (Garrocho-Villegas et al. 2008), and one gene encoding a putative haemoglobin-like protein HbO (LOC_Os06g39140). Notably, 4 out of the 5 genes coding for ns-Hbs (OsNSHB1-4) and the putative HbO-encoding gene were up-regulated in 24 h-treated roots only in Eurosis, suggesting a possible role of this class of molecules in the differential response of the two genotypes to the treatment. The ns-Hbs are localized in diverse plant organs, their expression is differently regulated under stress conditions, and their apparent function in vivo is to modulate the levels of ATP and nitric oxide (ns-Hbs class 1) or to facilitate the diffusion of O_2_ (ns-Hbs class 2) (Garrocho-Villegas et al. 2008), but the precise function of these genes in rice has not yet been revealed.

#### Photosynthesis-Related GO Terms

Surprisingly, in Loto treated roots, 11 enriched GO-terms were related to photosynthesis (Fig. 5c and d), namely, “chloroplast thylakoid membrane” (GO:0009535), “photosynthesis, light harvesting” (GO:0009765), “plastoglobule” (GO:0010287), “chloroplast” (GO:0009507), “photosynthesis” (GO:0015979), “thylakoid” (GO:0009579), “chlorophyll binding” (GO:0016168), “chloroplast envelope” (GO:0009941), “plastid” (GO:0009536), “photosynthetic electron transport chain” (GO:0009767) and “stromule” (GO:0010319). Nevertheless, this result is consistent with other literature data. For example, in a transcriptomic analysis of leaves and roots of rice seedlings subjected to acute dehydration, many photosynthesis-related GO terms were enriched in roots under dehydration, including GO:0009579, GO:0009765 and GO:0016168 (Minh-Thu et al. 2013). Moreover, a transcriptome analysis revealed a down-regulation of genes encoding Sigma70-like family proteins in rice roots under osmotic stress (Ma et al. 2009). These proteins are involved in the control of chloroplast gene expression. The authors suggested that root genes may regulate enzymes and proteins that are related to photosynthesis.

#### Cell Wall-Related Enriched GO Terms

Major differences between the leaf samples of the 2 genotypes were found at 24 h of stress treatment, where Eurosis was mainly characterized by GO terms related to cell wall-related processes and Loto by GO terms related to peroxisomes and photoinhibition (Fig. 6b and d). In the cell wall-related GO terms that were enriched in Eurosis, several genes associated with lignin polymerization were present, including many peroxidases at 3 h of treatment and several laccases both at 3 and 24 h of treatment. Increased lignification has been observed in the leaf elongation zone of drought-stressed maize leaves (Vincent et al. 2005) and increased cell wall peroxidase activity has been implicated in the cessation of leaf growth under drought in darnel (Bacon et al. 1997). Moreover, several genes, which were listed in these GO terms, encode xyloglucan-modifying enzymes; this finding is consistent with the observation that more xyloglucan is synthesized during dehydration, promoting the strengthening of the cell wall through cross-linking and tightening (Moore et al. 2008). Similar results were reported in a recent transcriptome profiling study of the leaf elongation zone under drought that compared the gene expression between drought-tolerant and drought-sensitive rice varieties (Cal et al. 2013). Many of the differentially expressed genes were involved in secondary cell wall deposition, lignin polymerization, or coded for glycosyl hydrolases and xyloglucan endotransglucosylase/hydrolases, suggesting that the two genotypes have alternative strategies for the regulation of leaf elongation under drought (Cal et al. 2013). Moreover, drought tolerance involves a restructuring of the cell wall that allows growth processes to occur at a lower water level; thus, cell wall adjustment under water stress is an important phenomenon in plant adaptation. These mechanisms, however, are complex and differ among plant species; many studies are still needed to understand how these processes influence plant stress tolerance (Moore et al. 2008).

### MapMan Pathway Analysis of 3 h-Treated Roots

**Fig. 7:**
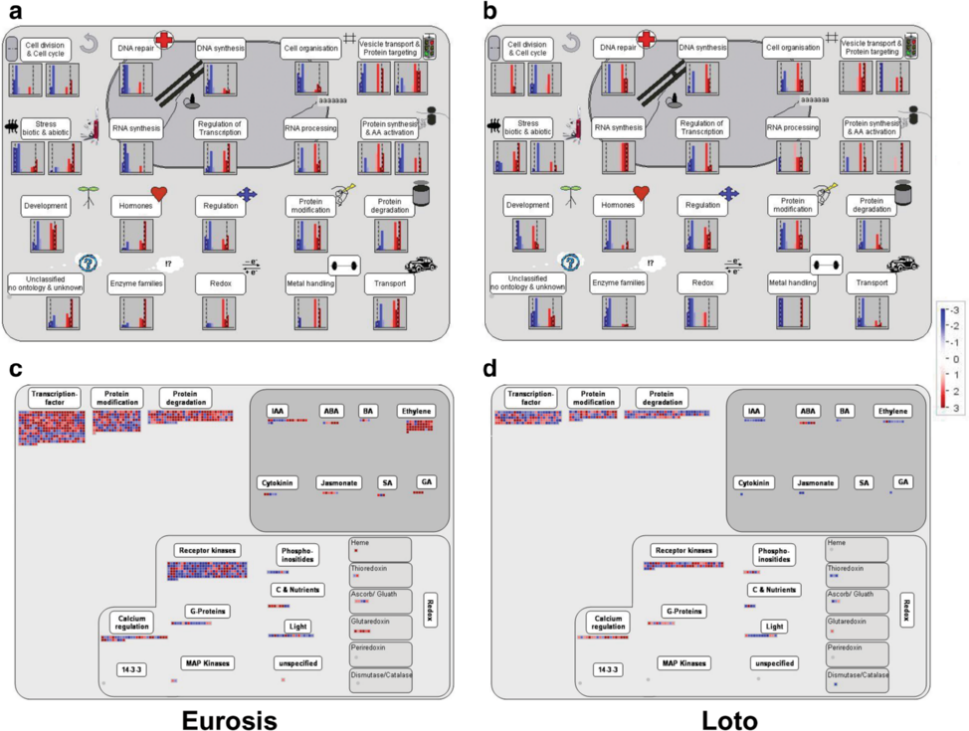
MapMan analysis of genotype specific up- and down-regulated genes in 3 h-treated roots. MapMan analysis of Eurosis (*left*) or Loto (*right*) DEGs in 3 h-treated roots. **a**, **b** Cell functions overview; **c**, **d** Regulation overview. The colour scale indicates the degree of fold change expression values (*light blue-blue, down-regulation; pink-red, up-regulation*)

Pathway-based analysis helps to further understand the biological function of genes. We used the MapMan package (http://mapman.gabipd.org/) as a tool to more thoroughly visualize the pathways involved in the osmotic stress response of the two genotypes. Because major differences in the stress response between the 2 cultivars were found in 3 h-treated roots, all of the DEGs specifically modulated in 3 h-treated root samples of Eurosis or Loto (3455 and 1410 genes in Eurosis and Loto, respectively; Table 3, Additional file 8: Table S8) were analysed using MapMan to identify differences in metabolic and regulatory pathways. The analysis showed that some pathways related to cell division, stress response, hormones, regulation, enzyme families and transport were differently regulated between the 2 cultivars, confirming the major results of the GO enrichment analysis (Fig. 7). In particular, the gene sets specific to each genotype included genes that encoded TFs, proteins involved in ethylene signalling and enzymes belonging to several families with a role in the plant stress response (e.g.*,* cytochrome P450; Additional file 9: Table S9).

Among TF-encoding genes, approximately 40 genes were up-regulated only in Eurosis (Additional file 9: Table S9). Among them, LOC_Os02g22020 codes for a GARP TF, which regulates the response to osmotic stress in rice (Mito et al. 2011). This gene is the putative orthologue of *HRS1*, which acts as a negative regulator of ABA signalling during Arabidopsis seed germination. HRS1 may participate in the suppression of ABA signalling in the germinating embryo axis, which in turn promotes the germination of Arabidopsis seeds in either normal or salt stress environments (Wu et al. 2012). Very recently, it has been proposed that HRS1 represses primary root growth in response to phosphorus deficiency, integrating the signalling pathways related to phosphate and nitrate (Medici et al. 2015).

It is well known that MYB TFs have a prominent role in the response to drought in plant species (Baldoni et al. 2015). Among the five *MYB* genes (LOC_Os01g34060, LOC_Os01g65370, LOC_Os05g48010, LOC_Os06g43090 and LOC_Os08g33150) that were up-regulated only in Eurosis (Additional file 9: Table S9), the LOC_Os01g65370 gene is very close to *OsMYB2P-1*, which is associated with Pi starvation signalling and is involved in the regulation of root architecture (Dai et al. 2012). This TF may have a role in the root growth of Eurosis plants.

In addition to the originally identified role in defence signalling, WRKY TFs have a pivotal function in seed germination, flower development, senescence and the abiotic stress response (Tripathi et al. 2014). Three *WRKY* genes (LOC_Os01g09080/*OsWRKY107*, LOC_Os03g55080/*OsWRKY3* and LOC_Os08g29660/*OsWRKY69*) were up-regulated only in Eurosis (Additional file 9: Table S9). Consistently, *OsWRKY69* expression is up-regulated in a drought tolerant cultivar (Douradão) compared to a sensitive cultivar (Primavera) under water shortage conditions, suggesting that its action in the signalling of the drought response may contribute to the tolerant phenotype of rice genotypes (Silveira et al. 2015). Interestingly, *OsWRKY69* expression is regulated by ABL1, which is a rice basic region/leucine zipper motif TF involved in the ABA response, suggesting that OsWRKY69 may be involved in the signalling of the abiotic stress response (Yang et al. 2011).

In higher plants, ethylene is synthesized via two enzyme-catalysed steps from S-adenosyl-L-methionine (SAM). The 1-aminocyclopropane-1-carboxylic acid (ACC) synthase (ACS) catalyses the cyclization of SAM to ACC and subsequently, the ACC oxidase (ACO) catalyses the oxidative conversion of ACC to ethylene (Yang and Hoffman 1984). ACS and ACO are encoded by medium and small-sized gene families, respectively, and their expression is differentially regulated by various developmental, environmental, and hormone signals (Bleecker and Kende 2000; Chen and McManus 2006; Binnie and McManus 2009). Here, MapMan analysis highlighted that some *ACO* genes were differentially regulated between the two genotypes: LOC_Os03g64280 (*ACO1*) and LOC_Os08g30080 were up-regulated only in Eurosis, and LOC_Os06g37590 was down-regulated only in Eurosis, whereas LOC_Os03g63900 was down-regulated only in Loto (Additional file 9: Table S9). Literature data indicate that *ACO1* is involved in the accumulation of ethylene during submergence, and its activity may contribute to the initiation of adventitious root formation during submergence through the activation of epidermal cell death signalling (Steffens and Sauter 2009; Fukao and Bailey-Serres 2008).

Furthermore, MapMan analysis showed that 6 cytochrome P450 encoding genes (LOC_Os01g41810, LOC_Os02g47470, LOC_Os03g04660, LOC_Os05g25640, LOC_Os05g31740 and LOC_Os10g09110) were up-regulated only in Eurosis, 2 cytochrome P450 encoding genes (LOC_Os01g12750 and LOC_Os11g18570) were up-regulated only in Loto, and 2 cytochrome P450 encoding genes (LOC_Os01g72260 and LOC_Os10g12080) were down-regulated only in Loto (Additional file 9: Table S9). LOC_Os02g47470 and LOC_Os05g31740, which were up-regulated only in Eurosis, seem to be involved in submergence tolerance (Kottapalli et al. 2007; Jung et al. 2010). In particular, LOC_Os02g47470 encodes the ABA 8’-hydroxylase (OsABA8ox1), which catalyses the major regulatory step of the predominant pathway for ABA inactivation via the conversion of ABA to phaseic acid (Kushiro et al. 2004). The involvement of *OsABA8ox1* in the drought response has been previously reported; the induction of *OsABA8ox1* under a water-deficient condition significantly suppressed the elevation of ABA levels (Yazawa et al. 2012). Ethylene partially contributes to the reduction of ABA concentration in submerged rice by activating *OsABA8ox1* (Saika et al. 2007); our observation about the up-regulation of the *ACO1* gene in Eurosis suggests that in 3 h-treated roots of this genotype a higher level of ethylene was present, leading to the up-regulation of *OsABA8ox1. OsABA8ox1* is up-regulated after submergence in a rice genotype that is tolerant to a prolonged submergence (Jung et al. 2010). Since restriction of ROS production or removal of ROS during submergence and after desubmergence are critical factors for survival, the authors suggested that the activation of *OsABA8ox1* may contribute to reducing ROS, which are produced during ABA signalling (Kwak et al. 2003; Mittler and Blumwald 2015). A similar mechanism may occur in Eurosis and contribute to osmotic stress tolerance.

Moreover, two of the 10 cytP450 genes that were differentially expressed between Eurosis and Loto encode two enzymes involved in the phenylpropanoid pathway, namely, LOC_Os05g25640 and LOC_Os10g12080. LOC_Os05g25640, which was up-regulated only in Eurosis, codes for the cinnamic acid 4-hydroxylase (C4H), which transforms the cinnamic acid into P-coumaric acid at the beginning of the phenylpropanoid pathway. LOC_Os10g12080, which is also named *CYP98A15p* (Liu et al. 2010), was down-regulated exclusively in Loto and encodes coumarate 3-hydroxylase (C3H), which transforms the P-coumaric acid into caffeic acid at the beginning of the lignin branch. An expression analysis conducted on two rice cultivars under oxidative stress showed a down-regulation of *CYP98A15p* in the more sensitive cultivar, which is consistent with our results (Liu et al. 2010). The authors hypothesized that the repression of lignin synthesis in the sensitive genotype may drive metabolic flux into flavonoids, which leads to a different metabolite flux between the tolerant and the sensitive genotypes (Liu et al. 2010). In a recent study, the expression level of genes involved in suberin and lignin production, including the *C4H* gene, correlates well with the absolute suberin and lignin content in rice roots (Ranathunge et al. 2015). Our observation of the up-regulation of *C4H* only in Eurosis and the down-regulation of *C3H* only in Loto suggests the presence of a higher amount of suberin and lignin in Eurosis roots. Similarly to the *C4H* gene, 4 above mentioned genes (e.g.*,* LOC_Os07g01020 encoding a putative SOR/SNZ family protein; LOC_OsOs03g06510 encoding a KRP; LOC_Os02g22020 encoding a GARP TF and LOC_Os01g65370 encoding a MYB TF) were differentially regulated between the analysed rice genotypes and were previously reported to be involved in root growth and development. These genes may have a role in the root growth and contribute to the tolerant response of Eurosis plants.

Recently, a transcriptomic analysis on Arabidopsis found that salt acclimation is mediated by DEGs involved in cell wall biosynthesis, osmoregulation and oxidative stress, by TF-encoding DEGs and by DEGs participating in the synthesis of lignin and ethylene biosynthesis (Shen et al. 2014). Here, we showed that similar categories of genes characterized the osmotic stress response of a tolerant rice genotype, suggesting that the tolerant phenotype of Eurosis to osmotic stress may be characterized by a similar mechanism involving the root system in particular.

## Conclusions

**Fig. 8:**
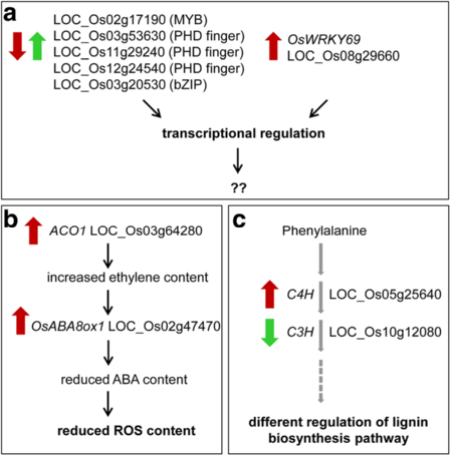
Schematic representation of some root mechanisms putatively involved in osmotic stress tolerance of Eurosis. *Red and green arrows* indicate gene regulation under osmotic treatment in Eurosis and Loto, respectively. *Grey arrows* indicate enzymatic steps in the lignin biosynthetic pathway

This study provided a comprehensive overview of the transcriptome changes that specifically occur in roots and leaves of two *japonica* rice genotypes, which were characterized by a contrasting phenotype in response to osmotic stress. RNA-Seq data highlighted that the tolerant genotype Eurosis and the sensitive genotype Loto mainly differed in their early response to osmotic stress in roots. A schematic representation of some mechanisms putatively involved in the higher tolerance to osmotic stress of Eurosis is shown in Fig. 8. Differential response included a prompt regulation of genes related to chromatin, cytoskeleton and transmembrane transporters in the tolerant genotype. Moreover, the differential regulation of TFs (Fig. 8a) and hormone-mediate signalling was observed. In particular, some of the hormone-related DEGs may reduce ROS content in the roots of the tolerant genotype (Fig. 8b). Furthermore, genes involved in the biosynthesis of lignin in the roots may determine the tolerant vs. sensitive response (Fig. 8c). The function of these genes in the water stress response and the physiological mechanisms in which they are involved still have to be elucidated. Nonetheless, some of the genes identified in this work may be important players in the tolerance response and could represent good candidate genes for improvement of the rice germplasm.

## Methods

### Plant Material, Growth Conditions and Osmotic Stress Treatment

Seventeen Italian rice cultivars (*O. sativa* L*.* ssp *japonica*) were subjected to PEG-mediated osmotic stress, namely Arborio, Asia, Augusto, Baldo, Carnaroli, Eurosis, Gigante Vercelli, Gladio, Koral, Loto, Maratelli, Salvo, SISR215, Thaibonnet, Venere, Vialone Nano and Volano. The rice seeds were kindly provided by the Rice Research Unit (CREA-RIS, Vercelli, Italy).

All of the hydroponic experiments were performed in a controlled growth room at 28 °C/23 °C under a 14 h light/10 h dark photoperiod with a light intensity of 200 μmol m^−2^ s^−1^ and 60 % relative humidity. Plant growth and osmotic stress treatment were performed as described in Baldoni et al. (2013). After 4 days of germination in Petri dishes, seedlings were grown in hydroponic culture with standard Yoshida nutrient solution (Duchefa Biochemistry, Harlem, The Netherlands). After 20 days of growth (third-leaf stage), half of the plants were transferred to a nutrient solution with 20 % (w/v) PEG6000 (Duchefa Biochemistry, Harlem, The Netherlands) at 11.00 am (3 h after the onset of illumination), to impose an osmotic stress treatment. For RNA purification, leaves (the second and third leaf) and roots from control or treated plants were collected separately in pools of 6 plants after 3 (14:00 pm) and 24 h (11:00 am) of PEG treatment.

### Physiological Measurements

Leaf RWC was measured after 0, 3, 24 and 48 h of PEG treatment, as described in Baldoni et al. (2013). Leaf EL was measured after 0, 3, 24 and 48 h of PEG treatment to evaluate cell membrane stability following the method of Shou et al. (2004) with minor modifications. For each sample, 6 leaves from 3 plants (2 leaves per plant) were collected, rinsed with deionized water and cut into 7-mm pieces. Leaf discs were incubated in tubes with 20 ml of deionized water, and the tubes were shaken overnight in a slanted position. The initial conductivity of the incubation (*C*i) was measured using a conductivity meter (Thermo Orion star Plus, Beverly MA) to estimate the amount of ions released from cells under normal conditions or PEG treatment. Leaf tissue in the incubation solution was then boiled at 100 °C for 30 min to completely disrupt the cell structure. The conductivity of the boiled solution (*C*max) was determined after cooling at room temperature. These 2 measurements were carried out individually for all of the samples from both the control and stressed plants. The percentage of EL was calculated by dividing the *C*i by the *C*max. For each genotype and condition, 7 biological replicates were measured.

For each sampling time, RWC and EL data were subjected to a one-way analysis of variance (ANOVA) after angular transformation of raw data. Comparison among means was performed using a Tukey’s test. Significant differences were accepted at *p* < 0.01.

### RNA Isolation and Quantitative RT-PCR Analysis

Total RNA was extracted from the leaves and roots using the TRIzol® RNA Purification Kit (Invitrogen, Carlsbad, CA) following the manufacturer’s instructions. RNA purity was checked spectrophotometrically (Nanodrop ND-1000 Spectrophotometer; Celbio, Italy), and only samples with a 260 ⁄ 280 nm ratio of absorbance comprised between 1.7 and 2.1 were further used. The integrity of the RNA was verified using the RNA 6000 Nano Labchip Kit on an Agilent 2100 Bioanalyzer (Agilent Technologies, USA), following the manufacturer’s protocol. Only samples with a 28S ⁄ 18S ratio ≥ 2 were used for further experiments.

The cDNA synthesis and quantitative RT-PCR was performed as described in Baldoni et al. (2013).

### Illumina RNA-Sequencing

Four micrograms of total RNA was subjected to library preparation using the TruSeq mRNA Sample Prep Kit from Illumina (Illumina, Inc., CA, USA) following the manufacturer’s instructions. Library concentration and size were assayed on a 2100 Bioanalyzer (Agilent). Single-end sequencing (50 bases) was conducted on an Illumina HiSeq2000 with samples run in 6-plex. Illumina sequencing was performed at IGA Technology Services Srl Service Provider (Udine, Italy).

### Bioinformatic Methods

#### Mapping of Illumina Reads

A preliminary read trimming (from 50 to 43 bases) step was performed to discard poor quality bases that were abundantly detected in the 3′ terminal region. The resulting trimmed reads were checked for contaminants, and low quality bases and contaminants were removed using the cutadapt software (Martin 2011). The spliced read mapper TopHat version 1.4.1 (Trapnell et al. 2012) was used to map reads to rice *O. sativa* Nipponbare genome (MSU release 6.16). A minimum and maximum intron length of 40 and 50,000 were used, respectively. Read counts were collected with HTSeq version 0.5.3 (Anders et al. 2014) in the single end and ‘union’ mode using *O. sativa* MSU 6.16 gtf file as performed for the ensemble plant repository.

#### Differentially Regulated Gene Calling

The DESeq Bioconductor package version 1.10.1 (Anders and Huber 2010) was used to call DEGs under R release 2.15.2. The cut-off for considering a gene as expressed was set to 0.1 RPKM (Reads per Kilobase per Million). Two distinct DESeq countSet object instances were created for Eurosis and Loto. DESeq parameters for dispersion estimation were the method ‘pooled’ and sharingMode ‘Maximum’. For DEG calling, the FDR threshold was set to 0.001 and the fold change threshold to 3.

#### Gene Ontology Enrichment Analyses

For goseq analyses, gene lengths were retrieved with BiomaRt queries (*O. sativa* MSU 6.16) out of rice Nipponbare cDNA, and the median length for each rice locus was obtained by parsing with R custom scripts. GO annotations were obtained using BiomaRt queries (*O. sativa* MSU 6.16). An FDR cut-off of 0.05 was used for GO enrichments.

#### Miscellaneous Bioinformatic Techniques

Heatmaps of the expression and various graphical outputs were generated with custom R scripts based on Bioconductor packages (Gentleman et al. 2004). Sample clustering was performed after data transformation via variance stabilizing transformation function (DESeq package; Anders and Huber 2010), and heatmaps were generated via the heatmap.2 function as available in the ‘gplots’ Bioconductor package. MapMan figures were generated upon binning of DEG sequences to MapMan bins by the Mercator application (Lohse et al. 2014). Unless otherwise stated, further graphical outputs were generated with custom R and Python scripts.

## Additional Files

Additional file 1: Table S1. Read number and alignment statistics. Passed-filter reads refer to preliminary Illumina inbuilt read scoring. (PDF 185 kb) Additional file 2: Table S2. Pearson correlation coefficients of samples. (XLSX 25 kb) Additional file 3: Table S3. Comparisons of RNA-Seq and qRT-PCR data. Comparisons of transcript fold changes as detected by RNA-Seq and qRT-PCR expression analyses for 10 selected genes in all of the experimental conditions. Comparisons of the differences between the means of treated and untreated samples of qRT-PCR data were performed using Student’s *t*-tests (*p-*value ≤ 0.05 except for the data marked with ^ns^). *OsCAT-A*: Catalase isozyme A; *OsZCO*: Zeaxanthin cleavage oxygenase. (PDF 291 kb) Additional file 4: Table S4. List of DEGs in all of the analysed samples. (XLSX 3592 kb) Additional file 5: Table S5. List of DEGs showing contrasting regulation between Eurosis and Loto samples. (XLSX 12 kb) Additional file 6: Table S6. List of common GO terms. List of GO terms common in all of the tissues and treatments, in all of the root samples or in all of the leaf samples. (XLSX 21 kb) Additional file 7: Table S7. List of enriched GO terms for the contrasts with accompanying enrichment *p*-values as resulting from goseq analysis. (XLSX 62 kb) Additional file 8: Table S8. List of DEGs specifically modulated in 3 h-treated root samples of Eurosis or Loto. (XLSX 184 kb) Additional file 9: Table S9. List of DEGs belonging to MapMan categories “Ethylene”, “Transcription Factor”, “Enzyme families - Cytochrome P450.” (XLSX 88 kb)

## Abbreviations

ABA: abscisic acid
ABC: ATP-binding cassette
ACC: 1-aminocyclopropane-1-carboxylic acid
ACO: ACC oxidase
ACS: ACC synthase
BP: biological process
C3H: coumarate 3-hydroxylase
C4H: cinnamic acid 4-hydroxylase
CC: cellular component
*C*i: initial conductivity
*C*max: absolute conductivity
DEG: differentially expressed gene
EL: electrolyte leakage
FDR: false discovery rate
GO: gene ontology
KRP: Kip-related protein
MATE: multidrug and toxic compound extrusion
MF: molecular function
ns-Hb: non-symbiotic haemoglobin
PEG: polyethylene glycol
RPKM: reads per kilobase per million
RWC: relative water content
SAM: S-adenosyl-L-methionine
TF: transcription factor
