# Supplementary material for: Comparative Leaf and Root Transcriptomic Analysis of two Rice Japonica Cultivars Reveals Major Differences in the Root Early Response to Osmotic Stress
Source: Rice (N Y). 2016 May 23;9:25. doi: 10.1186/s12284-016-0098-1 (PMC4877341; doi:10.1186/s12284-016-0098-1)
Supplement: Additional file 1: Table S1. — Read number and alignment statistics. Passed-filter reads refer to preliminary Illumina inbuilt read scoring. (PDF 185 kb) [file 12284_2016_98_MOESM1_ESM.pdf]

Sample	Passed-filter reads	Contaminant free reads	Total alignments	Mapped reads
Eurosis_03hCtr_F1	20763821	25234717	19868835	18407981
Eurosis_03hCtr_ F2	33696924	40967857	32355639	29984211
Eurosis_03hCtr_ F3	17336584	19567452	16622965	15605804
Eurosis_03hPEG_ F1	20575142	24367507	19778642	18510957
Eurosis_03hPEG_F2	25084404	28920392	24164646	22759615
Eurosis_03hPEG_ F3	24245826	28927119	23274367	21746953
Eurosis_24hCtr_ F1	20340311	24437597	19659712	18318909
Eurosis_24hCtr_ F2	21580630	24940146	20792859	19464850
Eurosis_24hCtr_ F3	21457278	24730114	20726381	19448144
Eurosis_24hPEG_ F1	36114436	42735816	34773252	32639947
Eurosis_24hPEG_ F2	26526461	31647671	25609379	23990573
Eurosis_24hPEG_ F3	17771368	20498371	17146859	16123550
Loto_03Ctrh_ F1	22513154	26334284	21676502	20230164
Loto_03hCtr_ F2	17469767	20250537	16702498	15617116
Loto_03hCtr_ F3	23188727	27276565	22350263	20866913
Loto_03hPEG_ F1	19610173	23247896	18953072	17820257
Loto_03hPEG_ F2	25145457	29562909	24298692	22862300
Loto_03hPEG_ F3	26069816	30244388	25246214	23837596
Loto_24hCtr_ F1	18563576	22224464	17912315	16682415
Loto_24hCtr_ F2	19896471	39029811	19190811	15927590
Loto_24hCtr_ F3	19927222	23214130	19220825	17970281
Loto_24hPEG_ F1	21819692	27219816	21046260	19626021
Loto_24hPEG_ F2	22269678	26682297	21402400	20072098
Loto_24hPEG_ F3	20604354	23844088	19775643	18615088
Eurosis_03hCtr_R1	20325938	22312285	18319464	16886959
Eurosis_03hCtr_R2	22181460	24688856	20268811	18666216
Eurosis_03hCtr_R3	33005934	35502802	30359756	28293045
Eurosis_03hPEG_R1	20640204	25866226	19459001	17398479
Eurosis_03hPEG_R2	22318856	25139003	20957762	19386020
Eurosis_03hPEG_R3	23964887	27674847	22740717	20934616
Eurosis_24hCtr_R1	25440063	27855286	23412699	21685208
Eurosis_24hCtr_R2	22821078	26520109	21298787	19461780
Eurosis_24hCtr_R3	30254897	32939393	28219288	26303241
Eurosis_24hPEG_R1	18804012	22297246	17434256	15820196
Eurosis_24hPEG_R2	21141453	23032199	19487417	18129823
Eurosis_24hPEG_R3	26559336	29058935	24615964	22884185
Loto_03hCtr_R1	32209705	37293510	30080240	27477074
Loto_03hCtr_R2	18625924	22999916	17519550	15725161
Loto_03hCtr_R3	34105327	43342752	32337433	28806745
Loto_03hPEG_R1	19153517	24442526	18349090	16406426
Loto_03hPEG_R2	23489512	26431380	21797838	20142504
Loto_03hPEG_R3	34038313	40669536	32012587	29179137
Loto_24hCtr_R1	21210047	25746111	18815511	16659239
Loto_24hCtr_R2	30157353	33251537	27638315	25451379
Loto_24hCtr_R3	30102568	31783085	26854970	24910883
Loto_24hPEG_R1	24513878	28591922	22727767	20749459
Loto_24hPEG_R2	23745922	30551365	22128820	19544509
Loto_24hPEG_R3	22398037	27654372	20435968	18225754
