# Supplementary material for: Comparative Leaf and Root Transcriptomic Analysis of two Rice Japonica Cultivars Reveals Major Differences in the Root Early Response to Osmotic Stress
Source: Rice (N Y). 2016 May 23;9:25. doi: 10.1186/s12284-016-0098-1 (PMC4877341; doi:10.1186/s12284-016-0098-1)
Supplement: Additional file 3: Table S3. — Comparisons of RNA-Seq and qRT-PCR data. Comparisons of transcript fold changes as detected by RNA-Seq and qRT-PCR expression analyses for 10 selected genes in all of the experimental conditions. Comparisons of the differences between the means of treated and untreated samples of qRT-PCR data were performed using Student’s t-tests (p-value ≤ 0.05 except for the data marked with ns). OsCAT-A: Catalase isozyme A; OsZCO: Zeaxanthin cleavage oxygenase. (PDF 291 kb) [file 12284_2016_98_MOESM3_ESM.pdf]

	Eurosis								Loto							
	**Roots 03 h**		**Roots 24 h**		**Leaves 03 h**		**Leaves 24 h**		**Roots 03 h**		**Roots 24 h**		**Leaves 03 h**		**Leaves 24 h**	
	**RNA Seq**	**qPCR**	**RNA Seq**	**qPCR**	**RNA Seq**	**qPCR**	**RNA Seq**	**qPCR**	**RNA Seq**	**qPCR**	**RNA Seq**	**qPCR**	**RNA Seq**	**qPCR**	**RNA Seq**	**qPCR**
**LOC_Os01g07120**<br>***OsDREB2A***	4.98	4.90	2.12	2.01	6.07	6.54	6.78	7.53	3.72	4.22	1.96	1.88 ^{ns}	5.86	8.47	5.51	4.96
**LOC_Os01g66120**<br>***OsNAC6***	29.49	16.68	3.77	1.96	9.89	7.99	8.71	7.12	8.59	9.54	8.16	7.52	9.55	9.50	15.20	12.33
**LOC_Os02g02400**<br>***OsCAT-A***	0.37	0.33	1.36	1.10 ^{ns}	0.32	0.42	0.28	0.33	0.19	0.39	2.73	2.40	0.16	0.24	0.18	0.22
**LOC_Os02g47510**<br>***OsZCO***	0.28	0.56	2.19	1.10 ^{ns}	0.20	0.30	0.08	0.08	0.06	0.18	0.42	0.50	0.16	0.20	0.04	0.03
**LOC_Os02g44870**<br>***OsDhn1***	10.27	4.17	0.58	0.53	21.03	24.20	18.42	17.80	11.01	8.15	0.96	1.11 ^{ns}	26.69	37.44	28.06	25.71
**LOC_Os03g20550**<br>***OsWRKY55***	4.11	4.23	0.86	0.90 ^{ns}	3.67	3.07	5.21	6.52	3.97	4.79	0.80	0.78 ^{ns}	3.22	5.03	8.13	8.33
**LOC_Os04g43680**<br>***OsMyb4***	16.26	14.14	3.36	2.72	6.90	9.12	11.44	15.15	8.01	11.74	2.60	3.45	6.66	10.03	26.47	31.57
**LOC_Os04g45810**<br>***OsHox22***	54.36	49.75	3.62	2.05	8.97	12.43	4.48	5.73	50.28	91.26	16.61	18.91	12.02	19.19	7.61	9.85
**LOC_Os05g25770**<br>***OsWRKY45***	11.54	9.71	0.94	0.76 ^{ns}	0.62a	0.65	0.32	0.39	5.24	7.85	1.00	0.96 ^{ns}	0.46	0.60	0.25	0.25
**LOC_Os10g33810**<br>***OsMyb8***	7.87	4.62	0.97	0.73 ^{ns}	8.65	10.16	29.97	49.14	4.11	4.73	1.20	1.20 ^{ns}	13.68	15.92	60.15	93.28
